# 2-Bromo-4-chloro-6-(cyclo­pentyl­imino­meth­yl)phenol

**DOI:** 10.1107/S1600536809039142

**Published:** 2009-10-03

**Authors:** Yu-Mei Hao

**Affiliations:** a Department of Chemistry, Baicheng Normal University, Baicheng 137000, People’s Republic of China

## Abstract

All atoms of the title mol­ecule, C_12_H_13_BrClNO, except the C and H atoms of the cyclo­pentane methyl­ene groups lie on a crystallographic mirror plane. The cyclo­pentane ring adopts an envelope conformation and an intra­molecular O—H⋯N hydrogen bond is observed. In the crystal, mol­ecules are stacked along the *b* axis by π–π inter­actions [centroid–centroid distance = 3.6424 (11) Å].

## Related literature

For the pharmaceutical and medicinal activity of Schiff bases, see: Dao *et al.* (2000 ▶); Sriram *et al.* (2006 ▶); Karthikeyan *et al.* (2006 ▶). For the coordination chemistry of Schiff bases, see: Ali *et al.* (2008 ▶); Kargar *et al.* (2009 ▶); Yeap *et al.* (2009 ▶). For the crystal structures of Schiff base compounds, see: Fun *et al.* (2009 ▶); Nadeem *et al.* (2009 ▶); Eltayeb *et al.* (2008 ▶). For bond-length data, see: Allen *et al.* (1987 ▶).
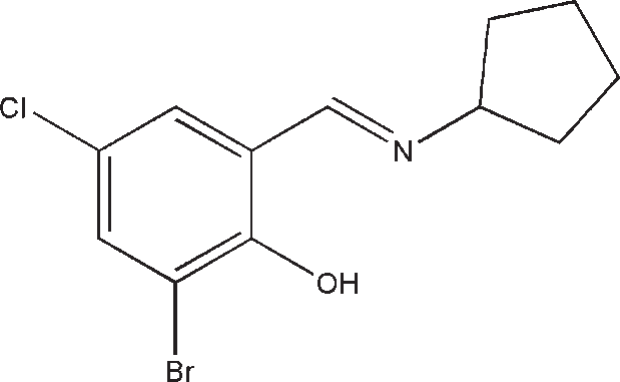


## Experimental

### 

#### Crystal data

C_12_H_13_BrClNO
*M* *_r_* = 302.59
Orthorhombic, 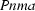

*a* = 12.142 (2) Å
*b* = 6.8610 (14) Å
*c* = 15.077 (3) Å
*V* = 1256.0 (4) Å^3^
*Z* = 4
Mo *K*α radiation
μ = 3.46 mm^−1^
*T* = 298 K
0.20 × 0.20 × 0.18 mm

#### Data collection

Bruker SMART CCD area-detector diffractometer
Absorption correction: multi-scan (*SADABS*; Sheldrick, 1996 ▶) *T*_min_ = 0.544, *T*_max_ = 0.574
10340 measured reflections
1488 independent reflections
1132 reflections with *I* > 2σ(*I*)
*R*_int_ = 0.040

#### Refinement

*R*[*F* ^2^ > 2σ(*F* ^2^)] = 0.044
*wR*(*F* ^2^) = 0.122
*S* = 1.08
1488 reflections
93 parameters
6 restraints
H atoms treated by a mixture of independent and constrained refinement
Δρ_max_ = 0.40 e Å^−3^
Δρ_min_ = −0.89 e Å^−3^

### 

Data collection: *SMART* (Bruker, 2002 ▶); cell refinement: *SAINT* (Bruker, 2002 ▶); data reduction: *SAINT*; program(s) used to solve structure: *SHELXS97* (Sheldrick, 2008 ▶); program(s) used to refine structure: *SHELXL97* (Sheldrick, 2008 ▶); molecular graphics: *SHELXTL* (Sheldrick, 2008 ▶); software used to prepare material for publication: *SHELXL97*.

## Supplementary Material

Crystal structure: contains datablocks global, I. DOI: 10.1107/S1600536809039142/ci2924sup1.cif

Structure factors: contains datablocks I. DOI: 10.1107/S1600536809039142/ci2924Isup2.hkl

Additional supplementary materials:  crystallographic information; 3D view; checkCIF report

## Figures and Tables

**Table 1:** Hydrogen-bond geometry (Å, °)

*D*—H⋯*A*	*D*—H	H⋯*A*	*D*⋯*A*	*D*—H⋯*A*
O1—H1⋯N1	0.89 (6)	1.71 (6)	2.577 (5)	162 (5)

**Fig. 1.**
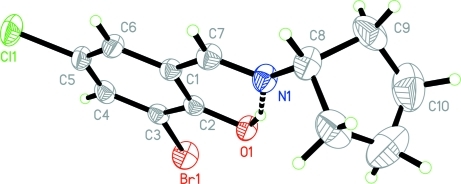
The molecular structure of the title compound, with 30% probability displacement ellipsoids. The dashed line indicates an intramolecular O—H···N hydrogen bond.

## supplementary crystallographic information

### Comment

Schiff base compounds are a class of important materials used in pharmaceutical and medicinal appications (Dao *et al.*, 2000; Sriram *et al.*, 2006; Karthikeyan *et al.*, 2006). Schiff bases have also been used as versatile ligands in coordination chemistry (Ali *et al.*, 2008; Kargar *et al.*, 2009; Yeap *et al.*, 2009). Recently, crystal structures of a large number of Schiff base compounds have been reported (Fun *et al.*, 2009; Nadeem *et al.*, 2009; Eltayeb *et al.*, 2008). In this paper, the title new Schiff base compound (Fig. 1) is reported.

All atoms of the title molecule, except the C and H atoms of the four methylene groups lie on a crystallographic mirror plane. The cyclopentane ring adopts a an envelope conformation. An intramolecular O—H···N hydrogen bond (Table 1) is observed. All bond lengths are within normal values (Allen *et al.*, 1987).

In the crystal, molecules are stacked along the *b* axis with π-π interactions [centroid to centroid distance = 3.6424 (11) Å].

### Experimental

3-Bromo-5-chlorosalicylaldehyde (0.1 mmol, 23.5 mg) and cyclopentylamine (0.1 mmol, 8.5 mg) were refluxed in a 30 ml methanol solution for 30 min to give a clear orange solution. Yellow block-shaped single crystals of the title compound were formed by slow evaporation of the solvent over several days at room temperature.

### Refinement

Atom H1 was located from a difference map and its positional parameters were refined. The remaining H atoms were constrained to ideal geometries, with C-H = 0.93–0.98 Å. The *U*_iso_(H) values were set at 1.2*U*_eq_(C) and 1.5*U*_eq_(O). The U^ij^ components of atom C10 were restrained to an approximate isotropic behaviour.

### Crystal data

**Table:**

C_12_H_13_BrClNO	*F*(000) = 608
*M**_r_* = 302.59	*D*_x_ = 1.600 Mg m^−^^3^
Orthorhombic, *P**n**m**a*	Mo *K*α radiation, λ = 0.71073 Å
Hall symbol: -P 2ac 2n	Cell parameters from 2255 reflections
*a* = 12.142 (2) Å	θ = 2.6–24.5°
*b* = 6.8610 (14) Å	µ = 3.46 mm^−^^1^
*c* = 15.077 (3) Å	*T* = 298 K
*V* = 1256.0 (4) Å^3^	Block, yellow
*Z* = 4	0.20 × 0.20 × 0.18 mm

### Data collection

**Table:**

Bruker SMART CCD area-detector diffractometer	1488 independent reflections
Radiation source: fine-focus sealed tube	1132 reflections with *I* > 2σ(*I*)
graphite	*R*_int_ = 0.040
ω scans	θ_max_ = 27.5°, θ_min_ = 2.2°
Absorption correction: multi-scan (*SADABS*; Sheldrick, 1996)	*h* = −15→15
*T*_min_ = 0.544, *T*_max_ = 0.574	*k* = −8→8
10340 measured reflections	*l* = −19→19

### Refinement

**Table:**

Refinement on *F*^2^	Primary atom site location: structure-invariant direct methods
Least-squares matrix: full	Secondary atom site location: difference Fourier map
*R*[*F*^2^ > 2σ(*F*^2^)] = 0.044	Hydrogen site location: inferred from neighbouring sites
*wR*(*F*^2^) = 0.122	H atoms treated by a mixture of independent and constrained refinement
*S* = 1.08	*w* = 1/[σ^2^(*F*_o_^2^) + (0.0628*P*)^2^ + 0.5702*P*] where *P* = (*F*_o_^2^ + 2*F*_c_^2^)/3
1488 reflections	(Δ/σ)_max_ = 0.001
93 parameters	Δρ_max_ = 0.40 e Å^−^^3^
6 restraints	Δρ_min_ = −0.89 e Å^−^^3^

### Special details

**Table:**

Geometry. All e.s.d.'s (except the e.s.d. in the dihedral angle between two l.s. planes) are estimated using the full covariance matrix. The cell e.s.d.'s are taken into account individually in the estimation of e.s.d.'s in distances, angles and torsion angles; correlations between e.s.d.'s in cell parameters are only used when they are defined by crystal symmetry. An approximate (isotropic) treatment of cell e.s.d.'s is used for estimating e.s.d.'s involving l.s. planes.
Refinement. Refinement of *F*^2^ against ALL reflections. The weighted *R*-factor *wR* and goodness of fit *S* are based on *F*^2^, conventional *R*-factors *R* are based on *F*, with *F* set to zero for negative *F*^2^. The threshold expression of *F*^2^ > σ(*F*^2^) is used only for calculating *R*-factors(gt) *etc*. and is not relevant to the choice of reflections for refinement. *R*-factors based on *F*^2^ are statistically about twice as large as those based on *F*, and *R*- factors based on ALL data will be even larger.

### Fractional atomic coordinates and isotropic or equivalent isotropic displacement parameters (Å^2^)

**Table:**

	*x*	*y*	*z*	*U*_iso_*/*U*_eq_	
Br1	−0.24144 (4)	0.2500	0.43777 (4)	0.0689 (3)	
Cl1	0.17429 (13)	0.2500	0.29606 (9)	0.0784 (5)	
O1	−0.1127 (2)	0.2500	0.60853 (19)	0.0495 (7)	
H1	−0.063 (5)	0.2500	0.652 (4)	0.074*	
N1	0.0610 (3)	0.2500	0.7067 (2)	0.0529 (9)	
C1	0.0712 (3)	0.2500	0.5489 (3)	0.0411 (9)	
C2	−0.0460 (3)	0.2500	0.5392 (3)	0.0401 (9)	
C3	−0.0878 (4)	0.2500	0.4537 (3)	0.0428 (9)	
C4	−0.0210 (4)	0.2500	0.3801 (3)	0.0510 (11)	
H4	−0.0517	0.2500	0.3235	0.061*	
C5	0.0921 (4)	0.2500	0.3908 (3)	0.0515 (11)	
C6	0.1374 (4)	0.2500	0.4740 (3)	0.0498 (10)	
H6	0.2136	0.2500	0.4804	0.060*	
C7	0.1190 (4)	0.2500	0.6365 (3)	0.0506 (10)	
H7	0.1953	0.2500	0.6418	0.061*	
C8	0.1145 (4)	0.2500	0.7942 (3)	0.0627 (14)	
H8	0.1950	0.2500	0.7893	0.075*	
C9	0.0723 (4)	0.0775 (6)	0.8482 (3)	0.0887 (14)	
H9A	0.0620	−0.0359	0.8107	0.106*	
H9B	0.1237	0.0447	0.8952	0.106*	
C10	−0.0323 (5)	0.1421 (10)	0.8850 (4)	0.133 (2)	
H10A	−0.0409	0.0934	0.9450	0.159*	
H10B	−0.0928	0.0934	0.8493	0.159*	

### Atomic displacement parameters (Å^2^)

**Table:**

	*U*^11^	*U*^22^	*U*^33^	*U*^12^	*U*^13^	*U*^23^
Br1	0.0544 (3)	0.0935 (5)	0.0588 (4)	0.000	−0.0150 (2)	0.000
Cl1	0.0888 (10)	0.0947 (11)	0.0517 (7)	0.000	0.0307 (7)	0.000
O1	0.0437 (16)	0.0639 (19)	0.0409 (16)	0.000	0.0048 (13)	0.000
N1	0.048 (2)	0.072 (3)	0.0388 (19)	0.000	−0.0052 (16)	0.000
C1	0.041 (2)	0.040 (2)	0.042 (2)	0.000	−0.0002 (16)	0.000
C2	0.049 (2)	0.032 (2)	0.039 (2)	0.000	0.0013 (17)	0.000
C3	0.051 (2)	0.035 (2)	0.042 (2)	0.000	−0.0035 (18)	0.000
C4	0.075 (3)	0.043 (2)	0.036 (2)	0.000	0.001 (2)	0.000
C5	0.063 (3)	0.049 (3)	0.042 (2)	0.000	0.015 (2)	0.000
C6	0.046 (2)	0.049 (2)	0.054 (3)	0.000	0.012 (2)	0.000
C7	0.041 (2)	0.061 (3)	0.051 (2)	0.000	−0.0026 (19)	0.000
C8	0.043 (2)	0.100 (4)	0.046 (2)	0.000	−0.010 (2)	0.000
C9	0.134 (4)	0.076 (3)	0.057 (2)	0.008 (3)	−0.034 (2)	0.007 (2)
C10	0.108 (4)	0.180 (6)	0.110 (4)	−0.023 (4)	0.015 (3)	0.040 (4)

### Geometric parameters (Å, °)

**Table:**

Br1—C3	1.881 (5)	C5—C6	1.370 (6)
Cl1—C5	1.743 (4)	C6—H6	0.93
O1—C2	1.322 (5)	C7—H7	0.93
O1—H1	0.89 (6)	C8—C9	1.526 (5)
N1—C7	1.271 (6)	C8—C9^i^	1.526 (5)
N1—C8	1.470 (5)	C8—H8	0.98
C1—C6	1.385 (6)	C9—C10	1.455 (7)
C1—C2	1.430 (6)	C9—H9A	0.97
C1—C7	1.443 (6)	C9—H9B	0.97
C2—C3	1.386 (6)	C10—C10^i^	1.480 (14)
C3—C4	1.374 (6)	C10—H10A	0.97
C4—C5	1.383 (7)	C10—H10B	0.97
C4—H4	0.93		
			
C2—O1—H1	99 (4)	N1—C7—H7	118.7
C7—N1—C8	120.1 (4)	C1—C7—H7	118.7
C6—C1—C2	119.6 (4)	N1—C8—C9	109.3 (3)
C6—C1—C7	120.8 (4)	N1—C8—C9^i^	109.3 (3)
C2—C1—C7	119.5 (4)	C9—C8—C9^i^	101.7 (5)
O1—C2—C3	120.7 (4)	N1—C8—H8	112.0
O1—C2—C1	121.9 (4)	C9—C8—H8	112.0
C3—C2—C1	117.3 (4)	C9^i^—C8—H8	112.0
C4—C3—C2	122.3 (4)	C10—C9—C8	105.1 (4)
C4—C3—Br1	118.9 (3)	C10—C9—H9A	110.7
C2—C3—Br1	118.8 (3)	C8—C9—H9A	110.7
C3—C4—C5	119.4 (4)	C10—C9—H9B	110.7
C3—C4—H4	120.3	C8—C9—H9B	110.7
C5—C4—H4	120.3	H9A—C9—H9B	108.8
C6—C5—C4	120.4 (4)	C9—C10—C10^i^	107.7 (3)
C6—C5—Cl1	121.4 (4)	C9—C10—H10A	110.2
C4—C5—Cl1	118.2 (3)	C10^i^—C10—H10A	110.2
C5—C6—C1	120.8 (4)	C9—C10—H10B	110.2
C5—C6—H6	119.6	C10^i^—C10—H10B	110.2
C1—C6—H6	119.6	H10A—C10—H10B	108.5
N1—C7—C1	122.7 (4)		
			
C6—C1—C2—O1	180.0	C4—C5—C6—C1	0.0
C7—C1—C2—O1	0.0	Cl1—C5—C6—C1	180.0
C6—C1—C2—C3	0.0	C2—C1—C6—C5	0.0
C7—C1—C2—C3	180.0	C7—C1—C6—C5	180.0
O1—C2—C3—C4	180.0	C8—N1—C7—C1	180.0
C1—C2—C3—C4	0.0	C6—C1—C7—N1	180.0
O1—C2—C3—Br1	0.0	C2—C1—C7—N1	0.000 (1)
C1—C2—C3—Br1	180.0	C7—N1—C8—C9	−124.7 (3)
C2—C3—C4—C5	0.0	C7—N1—C8—C9^i^	124.7 (3)
Br1—C3—C4—C5	180.0	N1—C8—C9—C10	−81.8 (4)
C3—C4—C5—C6	0.0	C9^i^—C8—C9—C10	33.7 (5)
C3—C4—C5—Cl1	180.0	C8—C9—C10—C10^i^	−21.6 (3)

Symmetry codes: (i) *x*, −*y*+1/2, *z*.

### Hydrogen-bond geometry (Å, °)

**Table:**

*D*—H···*A*	*D*—H	H···*A*	*D*···*A*	*D*—H···*A*
O1—H1···N1	0.89 (6)	1.71 (6)	2.577 (5)	162 (5)
